# Methodological challenges in measuring vaccine effectiveness using population cohorts in low resource settings

**DOI:** 10.1016/j.vaccine.2015.07.062

**Published:** 2015-09-11

**Authors:** C. King, J. Beard, A.C. Crampin, A. Costello, C. Mwansambo, N.A. Cunliffe, R.S. Heyderman, N. French, N. Bar-Zeev, for the VacSurv Consortium

**Affiliations:** a Institute for Global Health, University College London, 30 Guilford Street, London WC1N 1EH, United Kingdom; b London School of Hygiene and Tropical Medicine, London, United Kingdom; c Karonga Prevention Study, Karonga, Malawi; d MaiMwana Project Mchinji, Parent and Child Health Initiative, Lilongwe, Malawi; e Ministry of Health, Lilongwe, Malawi; f Institute of Infection and Global Health, University of Liverpool, Liverpool, United Kingdom; g Malawi-Liverpool-Wellcome Trust Clinical Research Programme, College of Medicine, University of Malawi, Blantyre, Malawi; h Liverpool School of Tropical Medicine, Liverpool, United Kingdom; i Division of Infection & Immunity, University College London, London, United Kingdom

**Keywords:** Survival analysis, Cohort, Methodology, Vaccine effectiveness, Children, Pneumonia, Diarrhoea, PCV13, RV1, Pneumococcal disease

## Abstract

Post-licensure real world evaluation of vaccine implementation is important for establishing evidence of vaccine effectiveness (VE) and programme impact, including indirect effects. Large cohort studies offer an important epidemiological approach for evaluating VE, but have inherent methodological challenges.

Since March 2012, we have conducted an open prospective cohort study in two sites in rural Malawi to evaluate the post-introduction effectiveness of 13-valent pneumococcal conjugate vaccine (PCV13) against all-cause post-neonatal infant mortality and monovalent rotavirus vaccine (RV1) against diarrhoea-related post-neonatal infant mortality. Our study sites cover a population of 500,000, with a baseline post-neonatal infant mortality of 25 per 1000 live births.

We conducted a methodological review of cohort studies for vaccine effectiveness in a developing country setting, applied to our study context. Based on published literature, we outline key considerations when defining the denominator (study population), exposure (vaccination status) and outcome ascertainment (mortality and cause of death) of such studies. We assess various definitions in these three domains, in terms of their impact on power, effect size and potential biases and their direction, using our cohort study for illustration. Based on this iterative process, we discuss the pros and cons of our final per-protocol analysis plan. Since no single set of definitions or analytical approach accounts for all possible biases, we propose sensitivity analyses to interrogate our assumptions and methodological decisions.

In the poorest regions of the world where routine vital birth and death surveillance are frequently unavailable and the burden of disease and death is greatest We conclude that provided the balance between definitions and their overall assumed impact on estimated VE are acknowledged, such large scale real-world cohort studies can provide crucial information to policymakers by providing robust and compelling evidence of total benefits of newly introduced vaccines on reducing child mortality.

## Highlights

• We discuss methodological challenges for evaluating vaccine effectiveness using cohorts.
• No single set of definitions or analytical approach can address all possible biases.
• Careful consideration of denominator, exposure and outcome definitions is needed.
• Sensitivity analyses are crucial to examine assumptions and explore subtle relationships.

## 1 Background

The 13-valent pneumococcal conjugate vaccine (PCV13) and monovalent rotavirus vaccine (RV1) were introduced to the routine infant vaccine schedule in Malawi in November 2011 and October 2012, respectively. Evidence of their effectiveness and population impact on mortality in sub-Saharan Africa is needed, particularly where HIV, malaria and malnutrition are prevalent. To date several modelling studies have projected their impact in this setting, but observational data on their empirically observed mortality impact, which exist elsewhere, are lacking for sub-Saharan Africa [1–7].

Pre-licensure vaccine efficacy is determined through placebo-controlled double-blind randomized trials [8]. Post-licensure studies are needed to determine vaccine effectiveness (VE) and population impact, including indirect effects (herd immunity and changes in transmission dynamics at the population level) in the ‘real world’ setting. Effectiveness is often assumed to be lower than efficacy, since cold chain implementation and stock administration are frequently suboptimal compared with strict trial conditions [9]. Measures of effectiveness are by nature observational and therefore vulnerable to confounding and bias, namely being unable to fully account for the individual decision to seek vaccination [10]. However, they may provide a more generalizable result, and because of size and exclusions, it is difficult for licensure trials to include an assessment of herd protection (unless randomized by cluster), a key benefit of many infant vaccines. Therefore, post-licensure effectiveness evaluations are crucial for policy makers to assess vaccine roll-out, highlight issues in programme implementation and determine total impact of direct and indirect effects at population level [8,11].

Several observational methods exist for evaluating VE: serological (using correlates of protection), ecological (population-level surveillance, including analysis of electronic medical records), cohort and case-control studies [8,12–17]. Each method has biases, advantages and disadvantages in practice, and variable utility in assessing potential non-specific vaccine benefits or risks. Each method also addresses slightly different questions about vaccine effectiveness and the preferred design may be context dependent; for example, a case-control design where the disease is extremely rare; or a cohort when investigating multiple outcomes for one exposure. Using a carefully selected and complementary combination of observational methods affords a more comprehensive understanding of vaccine impact, effectiveness and changing epidemiology of the target disease.

Cohort studies are resource intensive, requiring large sample sizes if events (such as death) are uncommon or if absolute effect sizes are small. As under-5 mortality rates are declining globally [18], cohorts with mortality end-points will become increasingly challenging. However, they avoid biases arising from selecting appropriate controls and censoring by survivorship to which other observational methods for estimating VE are more susceptible [19]. Three definitions are key to the design of a cohort study: the denominator (defining the study population), exposure ascertainment (with respect to vaccination status) and outcome ascertainment (mortality and cause of death) [20]. With these key parameters, the standard approach to analysis would be a comparison of the hazard of death or survival by vaccination status adjusted for key confounders.

In this paper, we aim to discuss key methodological challenges inherent to cohort designs focusing specifically on these three domains and apply them to our study setting to clearly illustrate practical considerations in establishing an *a priori* ‘per-protocol’ and sensitivity analysis plan. These analyses aim to give VE estimates, acknowledging that in complex field environments with unmeasured confounding (such as bias in the decision to receive vaccines or not), causality is difficult to assign.

## 2 Methods

We conducted a methodological review of cohort study design for evaluating vaccine effectiveness in a developing country setting. We searched Web of Science and PubMed using the following terms: method* AND/OR cohort, AND vaccine effectiveness AND/OR survival analysis, and included secondary references and highly-cited papers in the field. Our subsequent discussion focuses on the key challenges and considerations highlighted in the literature and use our field setting to illustrate key considerations. Based on this iterative process, we develop and present our primary and sensitivity analysis plan.

## 3 Cohort recruitment

### 3.1 Setting

A prospective cohort study is ongoing at two sites in Malawi: Mchinji district in the central region, and the Karonga demographic surveillance site (DSS) in northern Malawi [21,22]. Since 1 March 2012, we have conducted an open prospective cohort study in Mchinji district, which has a population of 465,000. The DSS site in Karonga has been running since 2002, covering a population of 35,000. Both sites are rural and the main occupation is subsistence farming [23]. Around 20% of the population is aged under 5 years and crude birth rate is approximately 40/1000 person years. Under-5 mortality has declined by 18 deaths/1000 live births over a 5 year period to a rate of 71/1000 in 2013 [24], with much of this effect seen in post-neonatal infants. The primary research questions for this cohort study are:

- What is the effectiveness of three doses of PCV13 against all-cause mortality in infants?
- What is the effectiveness of two doses of RV1 against diarrhoea-specific mortality in infants?

This study is being conducted alongside case-control studies with a range of morbidity and laboratory confirmed endpoints, to provide comprehensive data on vaccine effectiveness and impact in different population settings in Malawi [21].

### 3.2 Data collection

**Fig. 1:**
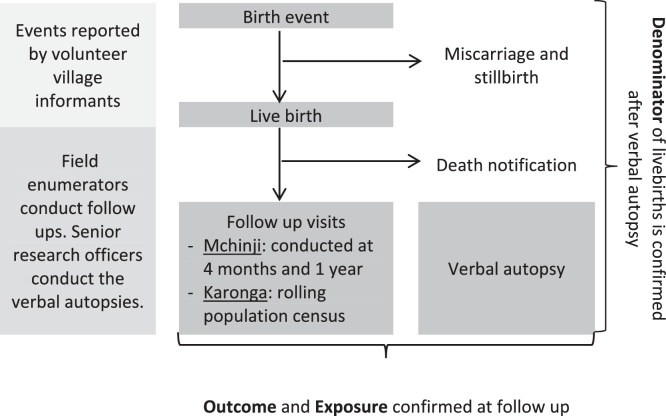
Schematic of study recruitment, follow up and definitions.

The DSS methods for Karonga have been published in detail previously [22]. The Mchinji surveillance site uses similar, but less resource-intensive methods, to the Karonga site [21]. Briefly, both systems are based on networks of volunteer village-level key informants who report monthly on births and deaths (Fig. 1). Selecting appropriate key informants was done with extensive community engagement to ensure they were acceptable to the community since without this support accurate reporting of events would be unlikely. In Mchinji infants are followed up by field enumerators with a home visit at 4 months and 1 year of age to capture vaccine status and confirm survival. At these visits, a one-page questionnaire is administered, collecting information on infant and mother survival, vaccine status (from documented health record or parental recall when documentation is unavailable), maternal education, household composition and assets. The large population size and human resource constraints limited the length of the questionnaire in the Mchinji setting. In the Karonga DSS, a rolling population demographic census visits all households in the entire population over the course of a year, survivorship and vaccine status are confirmed and extensive socio-economic questionnaires completed. In both sites, households with infant deaths are visited and a verbal autopsy (VA) questionnaire administered by senior research officers. Identical socio-economic and vaccine status questions are asked for surviving and deceased infants. The inclusion of two study sites, in addition to increasing sample size, allows one to act as an independent quality check on the other.

### 3.3 Sample size

For PCV13, we calculated the sample size based on the baseline assumption of a post-neonatal infant mortality of 25/1000 live births (reported by the Karonga DSS for 2011/2012), three-dose vaccine coverage of 75% and 12% loss to follow-up. Assuming vaccine can only lower and not increase mortality, we used a one-sided log-rank test to calculate that we require 34,848 infants surviving to 1 year and 729 deaths amongst post-neonatal infants (aged >28 days) to have 80% power to detect PCV13 vaccine effectiveness of ≥20% against all-cause mortality. Pneumonia, meningitis and sepsis account for 40% of deaths in post-neonatal infants, therefore ≥20% effectiveness was chosen as a modest estimate of effect size.

For RV1, our pre-RV1 introduction surveillance observed post-neonatal infant diarrhoea-related mortality of 6/1000 live births, and at 75% two-dose coverage and 12% loss to follow-up we require 43,668 infants and 210 diarrhoea deaths for 80% power to detect RV1 vaccine effectiveness of 36% against diarrhoea-related deaths. As we accrue more accurate information on cause-specific mortality and vaccine coverage, our targets may need revision. To date (May 2015), we have recruited 50,000 PCV13 eligible infants and 35,000 infants eligible for RV1.

## 4 Denominator

When defining the cohort sampling frame (”target population”), design decisions need to be made in terms of the age-eligibility and catchment population. Several age-eligibility options are possible, each with advantages and disadvantages with any cohort definition requiring a trade-off between power and estimated effect. Delayed timeliness in vaccine uptake means that inclusion from a younger age increases the number of partially vaccinated deceased infants, since they die before having the chance to receive all doses; this may overestimate VE as there are more deaths are in unvaccinated infants. However, using more liberal time cut-offs to allow for higher vaccine coverage would result in lower power (i.e. fewer recruited deaths) and misses a critical time period for deaths. We opted to include infants surviving to 14 weeks for the PCV13 analysis and 10 weeks for the RV1 analysis, following them to survival at 1 year, death or migration (censoring) before 52 weeks (Table 1). This definition maximizes cohort size, excluding only vaccine-age-ineligible infants. We will conduct sensitivity analyses using 6-week (earliest age-eligibility for vaccination) and 6-month (complete vaccination, allowing for poor timeliness) survival inclusion cut-offs, and 16 and 12 weeks for PCV13 and RV1, respectively (allowing for immunogenicity of the final dose).

We defined the eligible catchment population as all infants ‘born to an established household within the geographical region under surveillance’ (i.e. Mchinji District and the Karonga DSS). An ‘established household’ is defined as one which has been reported by village informants to have been present for at least 2 months at the time of the birth event. This seemingly straightforward demarcation is in reality more complex. For example, Mchinji district has two international borders, numerous commercial farming estates and neighbours the capital city district of Lilongwe which makes migration, resulting from seasonal harvesting and urbanization, challenging to monitor. Intricate situations (e.g. a complicated delivery being sent to a tertiary referral centre outside the district) may be misrepresented under this system, but such events are rare and unlikely to alter VE estimate substantially.

## 5 Exposure (vaccine status)

The exposure of interest is vaccination. Vaccination status can be ascertained from several sources, including care-giver report and documented health record, and the choice of source requires a trade-off between potential bias and power. Two key assumptions in the analysis of observational effectiveness studies are that there are no unmeasured systematic differences between the exposed and unexposed and that there is no differential exposure ascertainment by outcome [12]. These assumptions are likely to be violated, as outlined below.

### 5.1 Unmeasured differences between exposure groups

Vaccinated infants may differ in other health and social measures from unvaccinated infants. In our setting, households with lower incomes are more likely to have limited access to clean water and sanitation and by implication higher propensity to disease. When compounded by fewer available funds to seek care (and therefore vaccination, as has been shown in Karonga and elsewhere) [25–28] could result in higher mortality. Notably, limited care seeking is also a reason why community household surveillance is important, since such cases would be missed by healthcare-based surveillance. With the limited socio-economic information, we collect adequately accounting for the intricacies of the relationship between socio-economic factors, health status and health seeking behaviors is difficult. For these same reasons, knowledge of the birth and determining the vaccine status of infants who remain unvaccinated may be more challenging than ascertaining those who are subsequently vaccinated. Failure to identify unvaccinated infants would overestimate vaccine coverage among surviving infants resulting is a strongly biased increase in apparent vaccine effectiveness.

### 5.2 Exposure ascertainment by outcome

Many developing countries have three potential sources for ascertaining vaccine exposure: health passports, vaccine clinic registers and parental recall. In Malawi, children are issued a free ‘health passport’ at their first encounter with the healthcare system, in which vaccinations are recorded. All clinics that distribute vaccines are required to document delivered antigens in government implemented ‘Under-1 registers’, including information on each child's name, date of birth and village. At first vaccination, a non-unique sequential identifier is transcribed onto the health passport and is recorded at subsequent visits; in practice linkage across visits is poor and linkage between facilities impossible. The availability of health passports is associated with both exposure (e.g. infants with no health passport are less likely to have had any contact with the health system and therefore vaccinations) and outcome as the health passport is often buried with deceased infants. This violates the assumption that exposure ascertainment will not differ between deceased and surviving children.

Although relying on documented evidence of vaccination to determine exposure has a high positive predictive value (PPV) for true vaccination status, it may have poor negative predictive value (NPV). Missing documentation of vaccine receipt in surviving children (misclassification bias), would result in apparent decreased vaccine effectiveness as those truly vaccinated are recorded as unvaccinated. In deceased infants, misclassification as non-exposed in those vaccinated would bias toward apparent increased vaccine effectiveness [8]. As a quality control activity, we collect data on vaccine discrepancy between care-giver reports and health passports immediately following vaccination. Our data show 4% misclassification of vaccines, with 2% undocumented and 2% documented when not given, using care-giver report as the reference standard. Since the misclassification is randomly bi-directional, poor reporting is unlikely to introduce bias when documentation is available.

Parental recall is generally considered less reliable than documented evidence, as care-givers may over-report vaccination to meet expectations of health researchers or under-report if poorly recalled or parents hope their child will receive benefit of another dose. Evidence for the direction of such bias is limited; though a study from Kenya found parental recall underestimated vaccine coverage compared with documented status [29]. Classifying children according to parental report in the absence of documentation mitigates the biases of relying solely on documented evidence, which is less frequently available for deceased infants [30], but on the other hand may reduce the NPV. We will use combined data from the three sources to classify exposure ascertainment, applying a data reliability hierarchy to achieve the best balance between PPV and NPV (see Supplementary Material 1).

### 5.3 Multiple doses

Vaccination status can be defined as:Unvaccinated–received no doses; fully vaccinated–received all scheduled doses; partially vaccinated.

a. Non-outstanding: received at least one scheduled dose and not yet age-eligible for more.
b. Outstanding: received at least one scheduled dose, but age-eligible for further doses.

Including partially vaccinated infants into the vaccinated group could underestimate effectiveness if immune protection is partial. Including such infants in the unvaccinated group may result in children with partial protection being considered unvaccinated, again lowering estimates of vaccine effectiveness [8]. We define exposure based on doses received keeping partially vaccinated infants in the analysis as a discrete category; however our primary analysis will only include outstanding partially vaccinated infants.

Not distinguishing between outstanding and non-outstanding partially vaccinated infants could introduce bias in complex ways. Outstanding children are older and their delayed vaccination may be associated with other risk factors for poor outcome (such as poor health seeking behaviors); contrarily, an older living cohort introduces a survivor bias. By defining our cohort as infants surviving to age-eligibility for the final dose, we will not be including non-outstanding partially vaccinated infants. Sensitivity analyses using older and younger age cut-offs for inclusion will allow us to investigate further the effect of partially vaccinated infants.

### 5.4 Defining timeliness

**Fig. 2:**
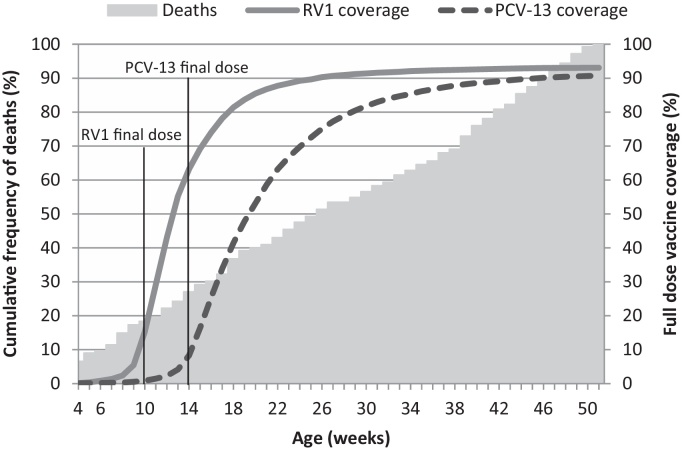
Vaccine timeliness and age at death in post-neonatal infants–example of cohort definition impacts on outcome and exposure.

Published timeliness definitions vary from strict (vaccination up to 14 days after recommendation), to lenient (receipt of vaccine up to 2 months delayed) [20,31–34]. Delayed vaccination is common [35,36]; in our data only 10% of infants received all recommended vaccines (BCG, four doses Polio, three doses Pentavalent and PCV13 and two doses RV1) within a fortnight of the schedule. Using a strict definition of timeliness would impact study recruitment (Fig. 2) and may introduce a bias as the low percentage of children who receive their vaccines according to the strict schedule may differ from the general population. On the other hand, including infants with a long delay may bias toward lower effectiveness. The timeliness definition is a trade-off between power and effect, strict definitions reducing power and lenient definitions reducing detectable effect. In the primary analysis, we will include timeliness of vaccine receipt as a covariate in the model, using the definition in Box 1 [35].

## 6 Outcome

Two key issues relating to outcome are ascertainment and definition, especially important for cause-specific mortality. Our study outcomes for PCV13 and RV1, respectively, are all-cause mortality in infants aged 14−52 weeks and diarrhoea-related mortality in infants 10−52 weeks.

### 6.1 Outcome ascertainment

To minimize differential censoring and misclassification, effort to ascertain outcomes should be equal in both outcome groups. Misclassification of deaths can occur for several reasons: inaccurate age at death, misunderstanding of death definitions, and unreliable reporting (e.g. relying on hearsay). Cultural barriers exist to openly discussing death in Malawi (especially among neonates, while in other cultures there may be a gender bias), leading to misclassification in reporting. Since death is uncommon more intensive resources are required for ascertaining and verifying death than survival. Under-ascertainment of deaths reduces both power and apparent effectiveness, as does misclassifying survivors as deaths. We conduct VA household visits on all under-five deaths, including stillbirths, to mitigate misclassification bias.

### 6.2 Cause-specific definitions

Like many developing countries [37], Malawi has no vital registration or death certification system outside of the hospital setting, therefore we use the WHO 2012 VA tool to collect data on signs and symptoms preceding death [38]. Interpretation of such data is the subject of vigorous debate [39]. Three options exist for analysis of verbal autopsies based on the WHO VA questions: physician review, custom-defined algorithm or standardized models such as InterVA (www.interva.net) or *InSilico*VA (arXiv:1411.3042). Physician review is resource intensive and has poor standardization, while automated methods are highly repeatable [39–42]. In the absence of a gold-standard, validity of cause-specific mortality attribution of any method is unknown.

Inherently non-specific, the all-cause mortality outcome for PCV13 lowers effect size, and our sample size calculation assumes a modest 20% reduction in deaths. Since there may be other drivers for reductions in infant mortality (e.g. improved nutrition status), the analysis would be confounded if these non-vaccine reductions are directly associated with individual vaccine status as well as mortality. However, we chose all-cause mortality due to its important policy implications and because syndromes commonly caused by *Streptococcus pneumoniae* (sepsis/meningitis, pneumonia) may be clinically non-specific and caused by other pathogens, leading to a more challenging outcome to capture accurately or consistently. We will conduct a sensitivity analysis for PCV13 against sepsis/meningitis or pneumonia deaths as defined by standardized models, and anticipate VE will be higher for these outcomes.

Compared with physician diagnosed diarrhoea-related mortality, study-specific algorithms have high sensitivity but poor specificity [43,44]. The latter may lower apparent VE. This bias arises from misclassifying non-diarrhoeal deaths as diarrhoeal or having a lower prevalence of rotavirus amongst selected deaths (e.g. during a cholera outbreak). Although false positives lower apparent effectiveness, with prevalent infections like rotavirus, the false positivity rate is lowered and the impact of the bias reduced [30]. Standardized computer models have shown variable performance when compared with physician review (likely a reflection of flaws in both techniques) [39–42]. We have decided to use a study-specific algorithm for diarrhoea-related mortality, and will conduct sensitivity analyses with standardized models. The all-cause mortality endpoint was abandoned for RV1 since PCV13 had been introduced in the previous year, further lowering post-neonatal infant mortality and resulting in unachievable power for that endpoint.

## 7 Primary analysis of vaccine effectiveness

To calculate the vaccine effectiveness of PCV13 and RV1, we will use Cox proportional hazards modelling of survival to 1 year [45]. Other reasonable analytical approaches (e.g. Mantel−Haenszel estimators, log−binomial regression, robust Poisson or negative binomial regression [46–48]) do not account for differential censoring. Cox modelling has two key assumptions: that censoring is unrelated to the outcome and that the hazards are proportional over time. For both assumptions, violations may occur in practice.

For example, itinerant agricultural laborers who leave the district for economic reasons may have a lower socio-economic status, which drives them to migrate, or harvesters who temporarily reside deep in the fields rather than at home may have lower likelihood of vaccination and independently higher mortality [26,49]. Therefore, if such families have higher infant mortality this violates the assumption that censoring is unrelated to outcome. The assumption of proportionality may also be violated as mortality risk (outcome) is generally age-associated, as is vaccination (exposure). Mortality in infants decreases with age while the likelihood of being vaccinated increases with each month survived, so there may be non-proportionality in outcomes between exposure groups over time. Methods for testing proportionality (e.g. Schöenfeld residuals, [50]) and mitigating its violations exist [51,52]. If we find the proportionality assumption violated, we will allow for time-varying covariates [53].

### 7.1 Confounders and risk factors

Since both vaccine receipt and outcome may be associated with classical confounding, measuring and adjusting for these is critical. In a Swedish study, unadjusted influenza VE was 50% while adjusted for confounders was 14% [54]; in our context, the magnitude and direction of bias are difficult to predict. We will include the following covariates in our primary analysis: socio-economic status; other vaccine receipt, timeliness and distance to nearest vaccination centre; mother's survival and health centre catchment (as a fixed variable). It is improbable that all confounders can be accounted for and differential ascertainment of confounders by exposure or outcome status is likely. For example, we collect mid-upper arm circumference (MUAC), a proxy measure of nutritional status in children and a good predictor of mortality [55]; collecting MUAC for surviving children is done at follow-up, but this is not possible for deceased children. In sensitivity analysis, we will investigate unmeasured confounding using a method described by Groenwold et al. [56], which simulates unmeasured confounders based on substantive knowledge of its association with the outcome and exposure.

## 8 Sensitivity analyses

The primary analysis of assessing individual vaccine status and survival adjusted for confounders and risk factors does not provide information on vaccine impact and is unlikely to account for all confounders or drivers of individual decisions in vaccine uptake. Therefore, the use of sensitivity analyses is essential for exploring indirect effects, the role of unmeasured confounders and confirming the findings of the primary analysis to provide a more complete picture of vaccine effectiveness.

### 8.1 Cluster-level analysis

Cluster-level analysis, either grouping by geographical region or by time-periods, addresses programme impact [12]. We will regress mortality rates against vaccine coverage at a geographical cluster level adjusting for socio-economic measures. In choosing the cluster definition (e.g. health centre catchment, community health worker catchment, village), we aim to maximize cluster number while maintaining adequate cluster size and inter-cluster heterogeneity. We will also investigate random-effects models that incorporate both individual and cluster-level covariates, which allow examination of cluster effects on individual mortality hazard.

### 8.2 Competing risks

A key consideration in carrying out survival analysis for cause-specific deaths is the issue of ‘competing risks’. A child dying of pneumonia cannot then die again of diarrhoea, and so is censored. Given the relatively high infant mortality rate in Malawi, such competing risks are an issue in the analysis of RV1 against diarrhoea-specific mortality endpoint. Competing risks regression will be used to investigate this, and may help explore synergistic effects of both vaccines [57].

### 8.3 Quasi-experimental approaches

Quasi-experimental approaches are used to evaluate causal inference from observational data, in an attempt to account for the lack of randomization in treatment assignment and therefore potential missed confounders (resulting in endogeneity). Two approaches which may be appropriate for vaccine effectiveness using cohort data are instrumental variables and propensity score matching. Using instrumental variables, a method widely used in econometrics, vaccine status would be replaced with a substitute exposure variable which is independent of the unmeasured confounder, thus avoiding endogeneity [58,59]. As socio-economic status may differ by exposure [26] and by outcome, it can give rise to a ‘self-selection bias’ whereby poor infants are both unvaccinated and die as a result of poverty, increasing apparent VE even after adjustment [49]. Using an instrumental variable could mitigate this bias and address any issues in missing exposure status, by selecting a variable associated with vaccination status but independent of socio-economic status. The challenge is selecting an appropriate instrumental variable. One possibility is vaccine stock availability at local clinic level. However, this too may be associated with socio-economic status of the catchment area, with clinics closer to tarmac roads both more likely to have regular supplies and more trade leading to higher socio-economic status of the surrounding population. An obvious choice for an independent instrumental variable is lacking in our situation, precluding the use of this method.

Propensity score matching is a method which accounts for the probability of receiving a treatment or intervention (i.e. vaccination) based on covariates to adjust the effectiveness model. This method has been used previously in the analysis of observational vaccine effectiveness studies [60,61], and could provide insight into our assumptions about residual unaccounted confounding.

## 9 Conclusion

The evaluation of vaccine effectiveness against mortality using cohort studies is complex and with globally declining mortality is becoming increasingly challenging as larger cohorts that are subject to more confounding are required. It is imperative to ensure that estimates of vaccine effectiveness are as valid and accurate as possible despite the biases inherent in large-scale, real-world observational studies. The complexities we have identified, such as differential vaccine ascertainment and lack of gold standard cause of death information, are likely to challenge vaccine evaluations in other low-resource settings and our approach to defining the primary analysis is generalizable to similar study designs.

While no single set of definitions or analytical approach can address all possible biases and confounding, the careful *a priori* consideration of denominator, exposure and outcome definitions and of the analytical approaches can achieve a balanced ‘per-protocol’ primary analysis (Box 2), which errs toward conservative estimation of vaccine effectiveness. Sensitivity analyses are crucial to interrogate assumptions and methodological decisions, and to explore more subtle relationships between mortality, vaccine exposure and inherent confounders. Only large-scale cohort studies can generate crucial evidence of total community mortality reduction from vaccines in resource poor settings, which lack routine vital registration and where mortality burden is greatest. Such studies provide essential information to policymakers by providing robust and compelling evidence of total benefits of vaccines on reducing mortality.

## Funding

This cohort studies were established through a Wellcome Trust Programme Grant (Number: WT091909/B/10/Z), the Wellcome Trust Core grants to the Malawi-Liverpool-Wellcome Trust, Blantyre, Malawi and to the Karonga Prevention Study, Chilumba, Malawi, and by a Wellcome Trust Strategic Award to University College London (Number: 085417ma/Z/08/Z).

## Conflict of interest

NBZ, NAC and NF have obtained investigator initiated project grant from GlaxoSmithKline Biologicals (GSK). NAC has received honoraria for participation in rotavirus vaccine advisory board meetings from GSK. All other authors declare no competing interests.

## Author contributions

NF, NAC, RSH, AC and CM designed the original study. CK, NBZ & JB conceived the paper. CK drafted the manuscript with significant contribution from NBZ and JB. All authors revised, edited and approved the final manuscript.

## Figures and Tables

**Table 1:** Summary of advantages and disadvantages of different target populations in a cohort study of vaccine effectiveness, using PCV13 as an example.

Age of inclusion	Target group	Advantages	Disadvantages	Direction of Bias	Comment
4–52 weeks	Post-neonatal infants	High recruitment of deceased infants	Includes vaccine ineligible infants	Overestimated VE	Includes infants who have not had the opportunity to be vaccinated (non-outstanding), therefore leading to higher vaccine effectiveness^a^
6–52 weeks	Any dose age-eligible infants	Relatively high recruitment of deceased infants	High proportion of unvaccinated infants	Overestimated VE
14–52 weeks	Fully vaccinated infants: age-eligible for final dose	Relatively high recruitment of deceased infants	Low vaccine coverage and protection	Unclear direction of bias	May decrease VE as vaccinated but unprotected infants are considered vaccinated. Or may increase VE as untimely vaccination leads to more unvaccinated infants being included
16–52 weeks	within 2 weeks of recommended week as a strict cut-off	Per guidelines definition of vaccination	Low vaccine coverage	Unclear direction of bias
18–52 weeks	within the recommended month as a moderate cut-off	Moderately high vaccine coverage	Low recruitment of deceased infants	Inconclusive VE	Using later cut-offs will decrease recruitment of deceased infants, reducing the power
20–52 weeks	within 6 weeks of the recommended month as a moderate cut-off	Moderately high vaccine coverage	Low recruitment of deceased infants	Inconclusive VE
26–52 weeks	by 6 months of age as a liberal cut-off	High vaccine coverage	Low recruitment of deceased infants	Inconclusive VE

PCV13: 13 valent pneumococcal conjugate vaccine; VE: vaccine effectiveness.

a In this scenario, infants who were not age-eligible for all doses but died before they had the opportunity to receive all doses would still be included in the analysis as either unvaccinated or partially vaccinated, increasing the apparent vaccine effectiveness.

## Box 1 Definition of vaccine timeliness.

We will use the following definition for defining timely vaccine receipt for PCV13 (3 doses) and RV1 (2 doses) [44]:

- Dose 1 (6 weeks) received between 4 weeks and 2 months.
- Dose 2 (10 weeks) received between 8 weeks and 4 months.
- Dose 3 (14 weeks) received between 12 weeks and 6 months.

We will also examine actual timeliness as a continuous covariate in sensitivity analysis

## Box 2 Per-protocol analysis definition.

Denominator: Children surviving to age-eligibility for the final dose (14 weeks for PCV-13 and 10 weeks for RV1), who were born to a household that had been continuously present in the geographical region under surveillance for at least 2 months prior to the birth, according to the village key informant.

Exposure: Either of documented evidence or parental report of vaccination status (assigned through a hierarchy algorithm), as a discrete numerical variable for each scheduled dose.

Outcome: All-cause mortality in children surviving to age-eligibility for the final dose confirmed by home visit (PCV13) *OR* pre-defined clinical algorithm of diarrhoea-associated mortality based on WHO 2012 VA questions in children surviving to age eligibility for the final dose (RV1).
