# Supplementary material for: Methodological challenges in measuring vaccine effectiveness using population cohorts in low resource settings
Source: Vaccine. 2015 Sep 11;33(38):4748–55. doi: 10.1016/j.vaccine.2015.07.062 (PMC4570930; doi:10.1016/j.vaccine.2015.07.062)
Supplement: Supplementary file 1 [file mmc1.docx]

Vaccine Assignment Hierarchy

As part of the evaluation of the 13-valent pneumococcal conjugate vaccine (PCV13) and monovalent rotavirus vaccine (RV1) on all-cause and cause –specific mortality, we are collecting vaccination status from surviving and deceased infants. There are several sources of where this information can be collected from:

- Health passports
- Caregiver recall
- Under 1 government vaccine registers

We ask to see health passports at all interviews (4 month surviving infants, 1 year surviving infants, verbal autopsies and rolling population surveillance). At the 1 year follow up interviews for surviving infants, we have over 90% health passport coverage and we consider this the ‘gold standard’ source of information (we are auditing the accuracy of health passport recorded vaccination status and finding a 4% bi-directional error rate).

In the deceased infants however, health passports are only found in about 15% of infants, and so we need to rely on other sources of vaccine information. How we combine these different sources could introduce errors or bias, so the following is a set of the rules for deriving vaccine status and the justifications for this approach. The reasoning for assigning different levels of reliability to different sources for deceased infants is summarized by the strengths and weaknesses of the sources in the table below.

| **Data Source** | **Strengths** | **Weaknesses** | **Reliability** |
| --- | --- | --- | --- |
| Health passport | Filled at the point of vaccination  Dates included  Less than 4% misreporting | Very few available | High |
| Health passport (4m) |  | Not complete cohort  Chance for subsequent vaccinations | High |
| Under 1 register | Routine data, therefore should be available for all | Some registers are missing or of very poor quality  Issues in tracing children through registers and across sites  Absence of record does not mean they are unvaccinated | Medium |
| Maternal report (date known) | Dates included  Generally some documented evidence provided | Uncommon | High |
| Maternal report (no vaccines) | Generally anecdotal support which makes it believable | Uncommon  Relies on accurate recall of events | High |
| Maternal report (yes/no) | Available for almost 100% of children | Recall can be biased (in both directions), so hard to adjust for the uncertainty  Chance of interviewer bias as interviewers may ‘interpret’ responses | Low |

**Figure 1: Hierarchy of data sources to assign vaccine status**

Less reliable sources account for about 70% of infants

Reliable sources account for about 30% of infants

Based on this hierarchy and the known issues within each source, the following rules will be applied in assigning vaccine status, and a binary variable will be created indicating ‘reliable/not reliable’ (sources in green and yellow considered reliable and in red not reliable). If a mixture of sources has been used to assign vaccine status, the record will be classified as reliable if all the PCV13 and RV1 vaccines come from a reliable source, and as not reliable if they are not.

- If a health passport is seen at verbal autopsy, this will be taken as correct
  - If a 4 month interview can be linked to the VA, all vaccines recorded at this point will replace those recorded unreliably at the VA. [Note: vaccines could have been received after the 4m interview, so only those recorded as being received at the 4m interview will be used to replace maternal report].
  - If vaccines have been recorded in the under 1 register, only those vaccines recorded will be used to replace those recorded unreliably at the VA. [Note: it is common for data to be missing from the registers, so only those positive indications of vaccination will be used].
  - If there is a conflict in data between data from the 4m interview, the under 1 register or maternal report, the information from the health passport at 4m will be taken as the correct, followed by the under 1 register and then maternal report.
